# Redox Control of Antioxidant and Antihepatotoxic Activities of* Cassia surattensis* Seed Extract against Paracetamol Intoxication in Mice: In Vitro and In Vivo Studies of Herbal Green Antioxidant

**DOI:** 10.1155/2016/6841348

**Published:** 2016-12-08

**Authors:** U. Seeta Uthaya Kumar, Yeng Chen, Jagat R. Kanwar, Sreenivasan Sasidharan

**Affiliations:** ^1^Institute for Research in Molecular Medicine (INFORMM), Universiti Sains Malaysia (USM), 11800 Pulau Pinang, Malaysia; ^2^Dental Research & Training Unit and Oral Cancer Research and Coordinating Centre (OCRCC), Faculty of Dentistry, University of Malaya, 50603 Kuala Lumpur, Malaysia; ^3^Nanomedicine-Laboratory of Immunology and Molecular Biomedical Research, Centre for Molecular and Medical Research, School of Medicine, Faculty of Health, Deakin University, Geelong, VIC 3216, Australia

## Abstract

The therapeutic potential of* Cassia surattensis* in reducing free radical-induced oxidative stress and inflammation particularly in hepatic diseases was evaluated in this study. The polyphenol rich* C. surattensis* seed extract showed good in vitro antioxidant.* C. surattensis* seed extract contained total phenolic content of 100.99 mg GAE/g dry weight and there was a positive correlation (*r* > 0.9) between total phenolic content and the antioxidant activities of the seed extract.* C. surattensis* seed extract significantly (*p* < 0.05) reduced the elevated levels of serum liver enzymes (ALT, AST, and ALP) and relative liver weight in paracetamol-induced liver hepatotoxicity in mice. Moreover, the extract significantly (*p* < 0.05) enhanced the antioxidant enzymes and glutathione (GSH) contents in the liver tissues, which led to decrease of malondialdehyde (MDA) level. The histopathological examination showed the liver protective effect of* C. surattensis* seed extract against paracetamol-induced histoarchitectural alterations by maximum recovery in the histoarchitecture of the liver tissue. Furthermore, histopathological observations correspondingly supported the biochemical assay outcome, that is, the significant reduction in elevated levels of serum liver enzymes. In conclusion,* C. surattensis* seed extract enhanced the in vivo antioxidant status and showed antihepatotoxic activities, which is probably due to the presence of phenolic compounds.

## 1. Introduction

There is currently much interest in the therapeutic potential of traditional and herbal therapy as an antioxidants in reducing free radical-induced oxidative stress and inflammation particularly in hepatic diseases [1]. The liver is the primary site for metabolism of almost all drugs because it is relatively rich in a large variety of metabolizing enzymes. Drug induced liver injury (DILI) is one of the most frequent causes of liver injury that poses a major clinical problem and challenge to drug regulators [2]. Drug induced liver injury makes up a total of 5% of all hospital admissions and 50% of all acute liver failures [3]. Paracetamol-induced hepatotoxicity has been linked with a number of cirrhosis, hepatitis, and suicide attempts cases. Paracetamol, if taken in overdose, can cause severe hepatotoxicity that leads to liver failure and nephrotoxicity depletion [4]. The toxic dose of acetaminophen caused the depletion of total glutathione (GSH) by as much as 90 percent, leading to accumulation of toxic metabolite N-acetyl-p-benzoquinone imine (NAPQI) which then covalently binds to cysteinyl sulfhydryl groups in hepatic protein through the 3-position of the benzene ring, forming NAPQI-protein adducts [5–7].

This causes the generation of ROS such as hydrogen peroxide (H_2_O_2_) and hydroxyl (OH^−^) radicals that affect the cellular membrane and induce lipid peroxidation by eliminating hydrogen from a polyunsaturated fatty acid and subsequent liver damage or necrosis [8–10]. The toxic metabolite formation and protein binding also cause the dysfunction of mitochondria that leads to adenosine triphosphate (ATP) depletion and oxidant stress [11, 12]. Moreover, numerous mitochondria are found in human liver cells, with about 1000–2000 mitochondria per cell, making up 1/5 of the cell volume [13]. Given the role of mitochondria as the cell's powerhouse, there may be some leakage of the high-energy electrons in the respiratory chain to form reactive oxygen species. This was thought to result in significant oxidative stress in the mitochondria with high mutation rates of mitochondrial DNA (mtDNA) [14]. A vicious cycle was thought to occur, as oxidative stress leads to mitochondrial DNA mutations, which can lead to enzymatic abnormalities and further oxidative stress. Therefore, liver cells with numerous mitochondria are found more vulnerable to free radical oxidation than any other cells in the body. Hence, medicinal plants with hepatoprotective activity are likely to make a considerable contribution for the liver protection against the paracetamol toxicity.

Medicinal plants are an important source of natural antioxidant agents because of the less toxic nature and being free from side effects compared to synthetic antioxidant [15, 16]. Most of these medicinal plants are rich in polyphenol which have the ability to scavenge-free radicals which are generated endogenously [17]. Polyphenols are not synthesized by human being and present only in plants and some microorganisms. Various studies exposed the antioxidant properties of polyphenols towards human pathologies [18].* Cassia surattensis *is one of the medicinal plants that is rich in polyphenol with health benefits. The genus* Cassia *is well known for its diverse biological and pharmacological properties, comprises about 600 species, and is vastly distributed worldwide [19]. The genus* Cassia *has been used as a potential medicinal plant since long ago [20, 21].* C. surattensis *belongs to the family Fabaceae, distributed throughout Malaysia, and is widely grown as ornamental plants in tropical and subtropical areas. This plant species has been traditionally used in many countries as food products and for medicinal uses. The bark and leaves of* C. surattensis* are said to be antiblenorrhagic [22]. The decoction of the roots [23] is commonly used to treat snake bites. The leaves are consumed for cough and sore throat and used for both internal and external cooling medicine.* C. surattensis* flowers and leaves have been studied extensively and the therapeutic properties such as antioxidant [24], antimicrobial [25], and antidiabetic [26] have been reported. According to Deepak et al. [27],* C. surattensis *seed showed good antioxidant, antifungal, and antibacterial activities on bacterial and fungal cultures. A finding by El-Sawi and Sleem [28] indicated the efficacy of* C. surattensis* leaf extract as hepatoprotective agent in CCl_4_-induced albino rats. Hence, present study is focused on redox control of antioxidant and antihepatotoxic activities of* Cassia surattensis* seed extract against paracetamol intoxication in mice. The outcome from this work may add to the overall therapeutic value of traditional and herbal medicine in hepatic diseases.

## 2. Materials and Methods

### 2.1. Plant Sample Collection

The matured pods of* C. surattensis* were collected from Universiti Sains Malaysia (USM), Pulau Pinang, Malaysia. The* C. surattensis* plant (leaves with flowers and pods) was authenticated by a botanist at the Herbarium of the School of Biological Sciences, Universiti Sains Malaysia, where a sample with voucher number 11464 has been deposited. The seeds were removed from the pods and were washed under running tap water to remove dirt prior to the drying process. The seeds were dried in an oven at 50°C. Then, the dried seeds were ground into powder and stored in airtight bottles.

### 2.2. Preparation of Plant Seed Extract

The powdered seeds (150 g) were soaked in methanol (500 mL) for 7 days under room temperature, 28°C [29]. The whole extract was filtered and methanol was evaporated from the filtrate by a rotary evaporator (Buchi, Switzerland) at 40–50°C to form a paste. Then, the extract was dried in the oven at 60°C to get a thick paste form. The crude extract was sealed in Petri plate and stored at room temperature, 28°C.

### 2.3. Total Phenolic Content

The total phenolic content of the extracts was determined using the method described by Li et al. [30]. One mL diluted Folin-Ciocalteau reagent was added to 1 mL of methanolic seed extract. Then, 4 mL sodium carbonate and 10 mL distilled water were added to the mixture. Subsequently, the mixture was allowed to stand 2 hours at room temperature, 28°C. The contents were centrifuged and absorbance was measured at 765 nm. The samples were prepared in triplicate for each analysis and the mean value of absorbance was obtained. The same procedure was repeated using various concentrations of gallic acid and a standard curve was constructed. Based on the measured absorbance, the concentration of phenolics was read (mg/mL) from the standard curve and the total phenolic contents of the extract were expressed as milligrams of gallic acid equivalents (GAE) per gram dry weight (mg GAE/g dw).

### 2.4. In Vitro Antioxidant Activity 

#### 2.4.1. Inhibition of 2,2-Diphenyl-1-picrylhydrazyl (DPPH) Radical Scavenging Assay

The DPPH radical scavenging activity of* C. surattensis* seed methanolic extract was carried out by previously described method by Sangetha et al. [24]. Five mL of a 0.004% (w/v) solution of DPPH in 80% methanol was added to 50 *μ*L of methanolic seed extract at different concentrations (0.078, 0.16, 0.31, 0.63, 1.25, 2.50, 5.00, and 10.00 mg/mL resp.). The reaction mixture was shaken vigorously. Butylated hydroxytoluene (BHT, Sigma) was used as a reference standard. The discoloration of DPPH was measured at 517 nm after 30 minutes incubation in the dark. The lower absorbance of the reaction mixture indicated higher free radical scavenging activity. All the tests were performed in triplicate.

The percentage DPPH radical scavenging was calculated using the following equation:

(1) $$\begin{array}{c} \%\;\;DPPH\;\;radical\;\;scavenging=\frac{A_{o}-A_{1}}{A_{o}}\times 100, \end{array}$$

where *A*_*o*_ is the absorbance of the control and *A*_1_ is the absorbance in the presence of the extract/standard.

#### 2.4.2. Inhibition of Nitric Oxide Radical Scavenging Assay

The assay was conducted based on the modification method by Chakraborthy [31]. The reaction mixture contained 1.5 mL sodium nitroprusside (10 mM) in phosphate buffer saline pH 7.4 and 0.5 mL of the seed extract at various concentrations (1.95, 3.91, 7.81, 15.63, 31.25, 62.50, 125.00, 250.00, 500.00, and 1000.00 *μ*g/mL) and incubated for 150 minutes at 25°C. Then, 1 mL of Griess reagent (1% sulphanilamide, 0.1% naphthylethylenediamine dichloride and 3% phosphoric acid) was added and the mixture was incubated for 30 minutes at room temperature, 28°C. The pink chromophore formed during diazotization of nitrite ions with sulphanilamide and subsequent coupling with naphthylethylenediamine dichloride was measured at 546 nm. The activity of seed extract was compared with ascorbic acid which was used as a reference standard. All tests were performed in triplicate. The nitric oxide radicals scavenging activity were calculated according to the equation:

(2) $$\begin{array}{c} \%\;\;Inhibition=\frac{A_{o}-A_{1}}{A_{o}}\times 100\%, \end{array}$$

where *A*_*o*_ is the absorbance of the control and *A*_1_ is the absorbance in the presence of the extract/standard.

#### 2.4.3. Reducing Power Assay

The reducing power assay was evaluated by the method of Oyaizu (1986) as described by Yildirim et al. [32]. One mL seed extract at various concentrations (0.02, 0.039, 0.078, 0.16, 0.31, 0.63, 1.25, 2.50, 5.00, and 10.00 mg/mL) was mixed with 2.5 mL phosphate buffer (0.2 M, pH 6.6) and 2.5 mL potassium ferricyanide [K3Fe(CN)6] (1% w/v). The mixture was incubated at 50°C for 20 minutes in a water bath. The reaction was stopped by adding 2.5 mL of trichloroacetic acid (TCA) solution (10% w/v) to the mixture and then centrifuged at 3000 rpm for 10 minutes. Then, 2.5 mL of the upper layer solution was mixed with 2.5 mL distilled water and 0.5 mL Ferric chloride solution (0.1% w/v). The reaction mixture was then incubated for 10 minutes at room temperature. Absorbance of the resultant mixture was measured at 700 nm. The increased absorbance of the reaction mixture indicated enhanced reducing power. Ascorbic acid was used as a reference standard.

#### 2.4.4. Calculation of Inhibition Concentration (IC_50_)

The IC_50_ is defined as the concentration of the sample that provides inhibition of 50% of the initial radical concentration with the unit, mg/mL or *μ*g/mL. The IC_50_ values were calculated from the linear regression plots of various concentrations of methanolic extract of* C. surattensis* seed/reference standard against the mean percentage of % inhibition obtained from three replicate tests.

### 2.5. In Vivo Antihepatotoxic Activities

#### 2.5.1. Animals

Fifteen adult male Swiss albino mice aged 6 to 8 weeks old and weighed 25 to 30 g were used to study the hepatoprotective activity of* C. surattensis* seed extract. The Animal Ethics Committee, Universiti Sains Malaysia, has approved the animal study for this project (USM/Animal Ethics Approval/2013/(90)(514)). The animals were kept under standard conditions (27 ± 2°C, relative humidity 44–56% and light and dark cycles of 10 hours and 14 hours, resp.) and fed with standard mice diet and purified drinking water ad libitum for 1 week before and during the experiments. The animals were obtained from Animal house of Universiti Sains Malaysia, Penang, and kept in cages under uniform husbandry condition, standard animal diet, and drinking water ad libitum. The food was withdrawn 18–24 hours before starting the experiment. All experiments were performed in the morning according to current guidelines for the care of the laboratory animals and the ethical guidelines for the investigation of experimental pain in conscious animals [33].

#### 2.5.2. Preparation of Paracetamol Dose Regimen and Treatments

The paracetamol tablets were obtained from a nearby pharmacy. Each tablet contains 500 mg of paracetamol. The mice were administered with paracetamol at a dose of 1 g/kg body weight (b.w.). The paracetamol was made into fine powder using a mortar and pestle. The powdered paracetamol was suspended in distilled water and was administered according to the body weight of mice. An aqueous suspension of seed extract was prepared in distilled water and different doses of* C. surattensis* seed extract (250 mg/kg b.w. and 500 mg/kg b.w.) and silymarin (200 mg/kg b.w.) were administered orally according to the body weight of mice [34].

#### 2.5.3. Mice Groupings and Treatments

Fifteen adult male Swiss albino mice were divided into 5 groups and each group consists of 3 mice each (Table 1). The pretreated normal control group received 10% dimethyl sulfoxide (DMSO) orally. The induced group was pretreated with 10% DMSO orally and given paracetamol once only (dose 1 g/kg b.w.) orally. The treatment Group I received orally both doses of 250 mg/kg b.w. of* C. surattensis* seed extract and 1 g/kg b.w. paracetamol, while the treatment Group II received orally both doses of 500 mg/kg bw of* C. surattensis* seed extract and 1 g/kg bw paracetamol, respectively. The positive control group was given silymarin at the dose of 200 mg/kg b.w. and paracetamol at the dose of 1 g/kg b.w. The mice in treatment and positive control groups were pretreated with the respective dose of seed extract/silymarin orally once daily for 7 consecutive days. Paracetamol dose at 1 g/kg b.w. was given to mice to induce hepatotoxicity. The oral administration of paracetamol was performed 3 hours after the last seed extract/silymarin administration on the 7th day except for the normal control group, which received only 10% DMSO. All mice were euthanized after 48 hours after paracetamol-induced hepatotoxicity [34].

#### 2.5.4. Biochemical Analysis

The mice of each group were anaesthetized with ketamine/xylazine and blood was collected directly from the heart. Then centrifuged at 3000 rpm for 15 minutes to separate the serum and kept at 4°C for analysis of various biochemical parameters including ALT, AST, and ALP [35]. All the analyses were performed using Hitachi 902 Automatic Analyzer using the adapted reagents from Roche (Germany) at Gribbles Pathology Laboratory Malaysia (M) Sdn. Bhd., Penang, Malaysia.

#### 2.5.5. Determination Body Weight and Relative Liver Weight

The mice were weighed daily during the study and the body weights of the mice were determined and recorded. After the mice were euthanized, the livers were isolated and washed with saline and weights were determined by using an electronic balance [36]. The liver weight was expressed with respect to relative liver weight. Relative liver weight was calculated using this formula:

(3) Relative  liver  weight  %=Liver  organ  weight×100Body  weight.

#### 2.5.6. Evaluation of the Antioxidant Status in the Liver Homogenate

Livers were perfused with saline and homogenized in chilled potassium chloride (1.17%) using a homogenizer to determine the in vivo antioxidant level in the liver tissues.

(*1) Glutathione (GSH) Activity Assay.* The GSH activity was quantified by using commercially available Glutathione Assay Kit (Sigma-Aldrich, USA). Initially, 10 mL of liver homogenate was added and mixed properly with 150 *μ*L of working solution consisting of 1.5 mg/mL DTNB, 6 U/mL glutathione reductase, and 1x assay buffer in a 96 well-plate before being incubated for 5 min. Subsequently, 50 mL of NADPH solution with a concentration of 0.16 mg/mL was added to each well in a 96 well-plate. Finally, the absorbance was measured by using an ELISA Plate Reader (Molecular Devices Inc., USA) at 412 nm wavelength at 1 min intervals for 5 min [37].

(*2) The Malonyldialdehyde (MDA) Assay.* Each liver homogenate (200 *μ*L) was diluted with 800 *μ*L of PBS and mixed with 25 *μ*L of 8.8 mg/mL butylhydroxytoluene and 500 *μ*L of 50% trichloroacetic acid. The mixture was vortexed, incubated for 2 h on ice, and centrifuged at 2000 ×g for 15 min. The supernatant (1 mL) was transferred into a new tube and mixed with 75 *μ*L of 0.1 M EDTA and 250 *μ*L of 0.05-M 2-thiobarbituric acid. The mixture was boiled for 15 min and allowed to cool to room temperature before the absorbance was measured at 532 and 600 nm in an ELISA Plate Reader (Molecular Devices Inc., USA) [37].

(*3) Super Oxide Dismutase (SOD) Assay.* Initially, a master mixture comprised 0.1 mol/L phosphate buffer, 0.15 mg/mL sodium cyanide in 0.1 mol/L ethylenediaminetetraacetic acid (EDTA), 1.5 mmol/L nitroblue tetrazolium, and 0.12 mmol/L riboflavin was prepared. Afterward, 200 *μ*L of master mixture was added to 100 *μ*L of serially diluted liver homogenates in a 96 well-plate before mixed thoroughly. Lastly, the absorbance was read by using an ELISA Plate Reader (Molecular Devices Inc., USA) at 560 nm wavelength and the SOD activity in the liver homogenate was expressed as units SOD/mg protein [37].

#### 2.5.7. Histopathological Observations

The liver samples of the mice were fixed in 10% buffered formalin. After fixation, the livers were dehydrated in a graded series of alcohol, cleared in xylene, and embedded in paraffin wax. Multiple 5 *μ*m sections from each block were mounted on slides. After staining with hematoxylin and eosin (H&E), slides were examined under a microscope for histopathological changes [34].

### 2.6. Statistical Analysis

Data are expressed as mean ± Standard Deviation (SD). Significance was evaluated using *t*-test and one-way ANOVA test (SPSS 13.0, SPSS Inc., Chicago, III) followed by Tukey post hoc multiple comparisons test for unpaired values. Regression analysis was performed to calculate the dose-response relation. Linear regression analysis was performed to find out the correlation coefficient. *p* < 0.05 was considered statistically significant.

## 3. Results

### 3.1. Extract Yield

The extraction was carried out using matured* C. surattensis* seeds. The weight of powdered seeds was 52.22 g. The weight of the seeds extract in paste form was 14.92 g. The extraction process yielded 28.57% of* C. surattensis* seeds extract.

### 3.2. Total Phenolic Content

The total phenolic content of the* C. surattensis* seed extract was expressed as mg gallic acid equivalent/g dry weight and calculated by using the gallic acid standard curve equation: *y* = 0.052*x* + 0.311 (*R* ^2^ = 0.992). The total phenolic content of* C. surattenis *seed extract was 100.99 mg GAE/g dry weight.

### 3.3. Radical Scavenging Activity

#### 3.3.1. 2,2-Diphenyl-1-picrylhydrazyl (DPPH) Radical Scavenging Assay

**Figure 1:**
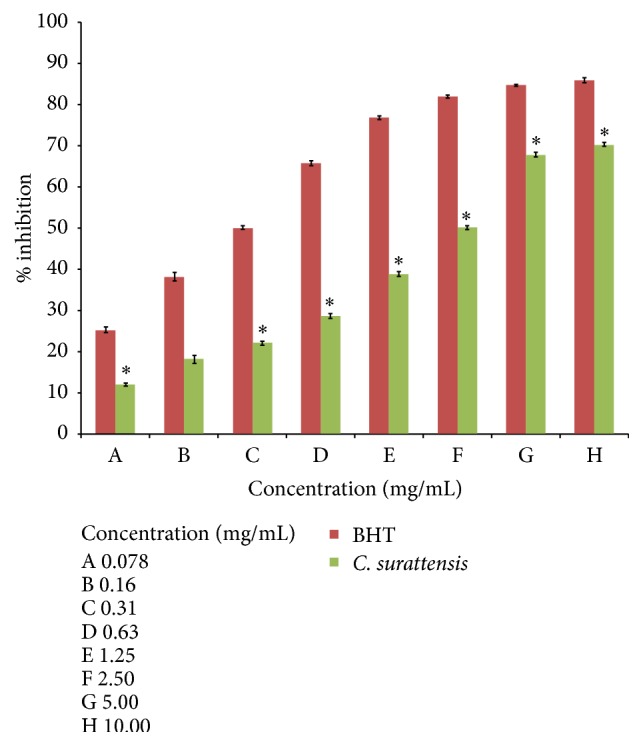
Percentage inhibition of methanolic seed extract of* C. surattensis* on DPPH free radicals compared to butylated hydroxytoluene (BHT). Each value is expressed as mean ± SD (*n* = 3), ^*∗*^ *p* < 0.05 compared with BHT.

**Figure 2:**
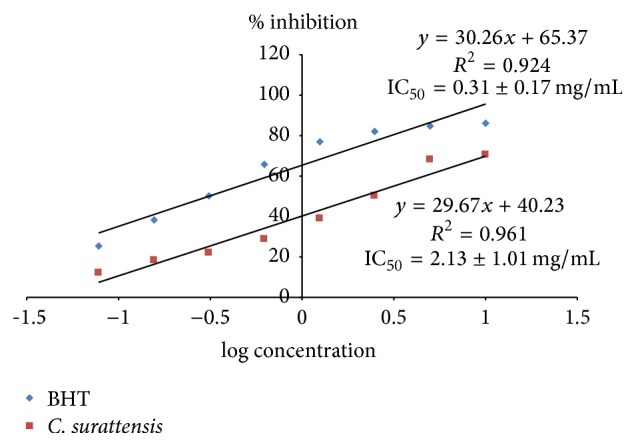
Inhibition effect of* C. surattensis *seed extract on DPPH free radicals compared with butylated hydroxytoluene (BHT).

Figures 1 and 2 show that the dose-response activity of DPPH radical scavenging activity of the methanol extract of the* C. surattensis* seed compared to the standard antioxidant BHT. The methanolic seed extract showed the highest scavenging activity, 70.39% ± 0.50 at 10 mg/mL, and lowest, 12.18% ± 0.35 at 0.078 mg/mL. The methanolic seed extract exhibited concentration dependent antioxidant activity by inhibiting DPPH radical with inhibitory concentration 50% (IC_50_) value of 2.13 ± 1.01 mg/mL and BHT was 0.31 ± 0.17 mg/mL (Figures 1 and 2).

#### 3.3.2. Nitric Oxide (NO) Radical Scavenging Assay

**Figure 3:**
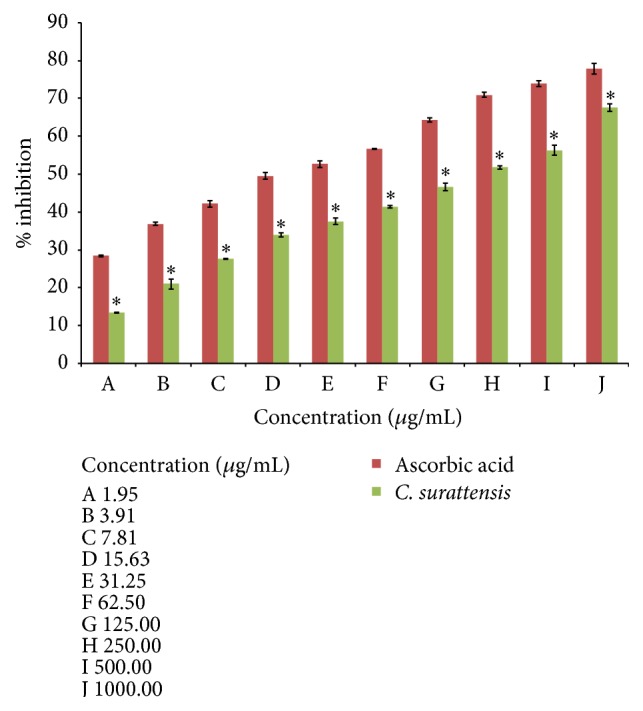
Percentage inhibition of methanolic seed extract of* C. surattensis* on nitric oxide radicals compared to ascorbic acid. Each value is expressed as mean ± SD (*n* = 3), ^*∗*^ *p* < 0.05 compared with ascorbic acid.

**Figure 4:**
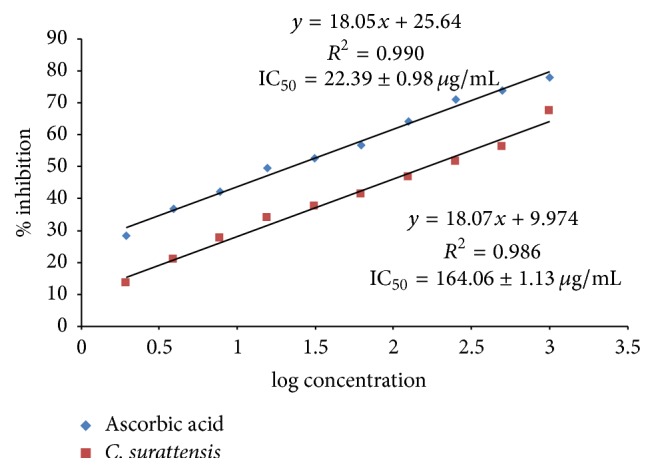
Inhibition effect of* C. surattensis *seed extract on nitric oxide radicals compared with ascorbic acid.

Dose-dependent NO scavenging activity of the methanol extract of the* C. surattensis* seed is shown in Figures 3 and 4. The methanolic seed extract showed the highest scavenging activity (67.60% ± 1.07) at 1000 *μ*g/mL and lowest (13.42% ± 0.13) at 1.95 *μ*g/mL. The methanolic seed extract of* C. surattensis* exhibited concentration dependent antioxidant activity by inhibiting nitric oxide radical with IC50 value of 164.06 ± 1.13 *μ*g/mL and ascorbic acid was 22.39 ± 0.98 *μ*g/mL (Figure 3). The IC50 values of the ascorbic acid were comparatively lower than seed extract which indicates higher antioxidant activity of ascorbic acid compared to the seed extract (Figure 4). Previously, Parul et al. [38] demonstrated that ascorbic acid has a strong antioxidant activity on the NO radical.

#### 3.3.3. Reducing Activity

**Figure 5:**
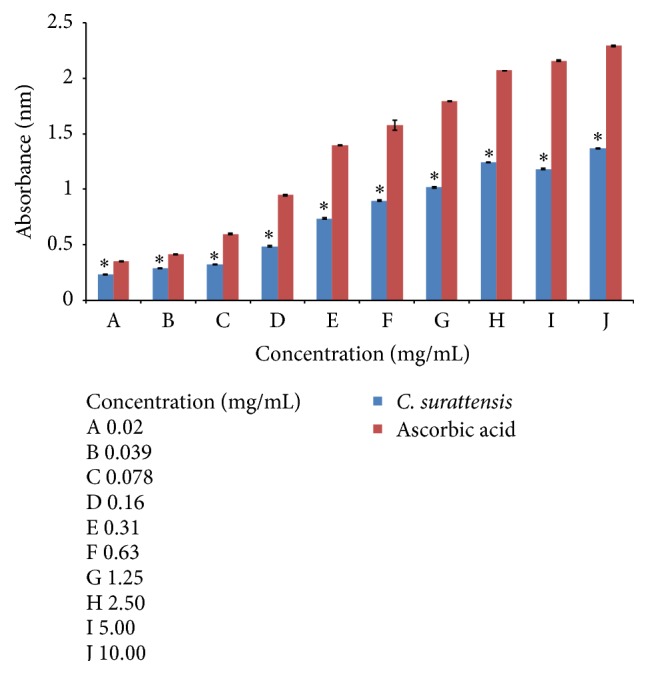
Reducing power of methanolic seed extract of* C. surattensis *compared to ascorbic acid. Each value is expressed as mean ± SD (*n* = 3), ^*∗*^ *p* < 0.05 compared with ascorbic acid.

The dose-response action for the reducing activity of methanol extract of* C. surattensis* seed is shown in Figure 5. The seed extract demonstrated reducing power activity in all the concentration tested, in a concentration-dependent manner. The seed extract reduced ferricyanide complex (Fe3+) to the ferrous form (Fe2+) and this showed that the seed extract has antioxidant properties. The methanolic seed extract showed the highest absorbance, 1.366 ± 0.0036 at 10.00 mg/mL, and lowest, 0.235 ± 0.0021 at 0.02 mg/mL. The higher absorbance of the reaction solution indicates the greater reducing power and greater antioxidant activity [39]. Ascorbic acid showed greater reducing power than that of the* C. surattensis* seed extract.

### 3.4. In Vivo Antihepatotoxic Activities

#### 3.4.1. Determination of Body Weight and Relative Liver Weight

Table 2 shows that the average body weights of the experimental animals were not affected by paracetamol, silymarin, and* C. surattensis* seed extract. The paracetamol administration caused a significant increase in the average liver weight of the paracetamol-induced group compared to the negative control group. The pretreatment with* C. surattensis* seed extract, at doses of 250 and 500 mg/kg b.w., and silymarin at a dose of 200 mg/kg bw significantly reduced the increased liver weight in paracetamol-induced group. A significant elevation of relative liver weight was seen in paracetamol-induced group, 8.15 ± 0.35%, when compared to the negative control group, 5.82 ± 0.31%, indicating the paracetamol-induced hypertrophy of these tissues. By contrast,* C. surattensis* seed extract, at dose of 250 mg/kg bw and 500 mg/kg bw, and silymarin at a dose of 200 mg/kg bw in combination with paracetamol significantly (*p* < 0.05) reduced the value of the relative liver weights to 5.99 ± 0.18%, 6.03 ± 0.47%, and 6.12 ± 0.41%, respectively, suggesting the possibility of* C. surattensis* seed extract to give protection against liver injury upon paracetamol administration.

#### 3.4.2. Biochemical Analysis

The effect of* C. surattensis* seed extract on liver marker enzymes (ALT, AST, and ALP) is displayed in Table 3. The data exhibited that the negative control group demonstrated a normal range of ALT, AST, and ALP levels. However, paracetamol administration caused a significant elevation in the ALT, AST, and ALP levels to 1689 ± 102.14 U/L, 2998 ± 189.22 U/L, and 341.51 ± 38.11 U/L, respectively, compared to the negative control group with pretreated 10% DMSO. A single oral dose of paracetamol at 1 g/kg bw caused a drastic increase in the serum liver marker enzyme activities of ALT, AST, and ALP [40], indicating an acute hepatotoxicity induced by administration of paracetamol. According to the Table 3 data, the biochemical parameters of the* C. surattensis* seed extract pretreated group were greater than those of the negative control group (*p* < 0.05), but it showed much lower levels of ALT, AST, and ALP than the paracetamol-induced group; that is, the extract treatment significantly reduced the previously elevated levels of ALT, AST, and ALP in liver tissue of hepatotoxic mice.

#### 3.4.3. The Antioxidant Status in the Liver Tissues

The in vivo antioxidant level in the liver tissues of paracetamol-intoxicated mice pretreated with* C. surattensis* seed extract was evaluated by various antioxidant assays, namely, GSH assay, SOD assay, and MDA assay (Table 4). The data exhibited that the negative control group revealed a normal range of GSH, SOD, and MDA levels, while the paracetamol-treated group displayed elevated levels of MDA and with decreased level of GSH and SOD, approving that paracetamol triggered liver injury at higher doses. However, the* C. surattensis* seed extract or silymarin pretreatment significantly elevated the previously dropped levels of GSH and SOD which led to depletion of the MDA levels in liver tissue. These findings clearly demonstrating the in vivo antioxidant activity* C. surattensis* seed extract at the dose of 250 mg/kg and 500 mg/kg by significantly reversing (*p* < 0.05) the effect produced by the paracetamol triggered liver injury.

#### 3.4.4. Histopathological Observation

**Figure 6:**
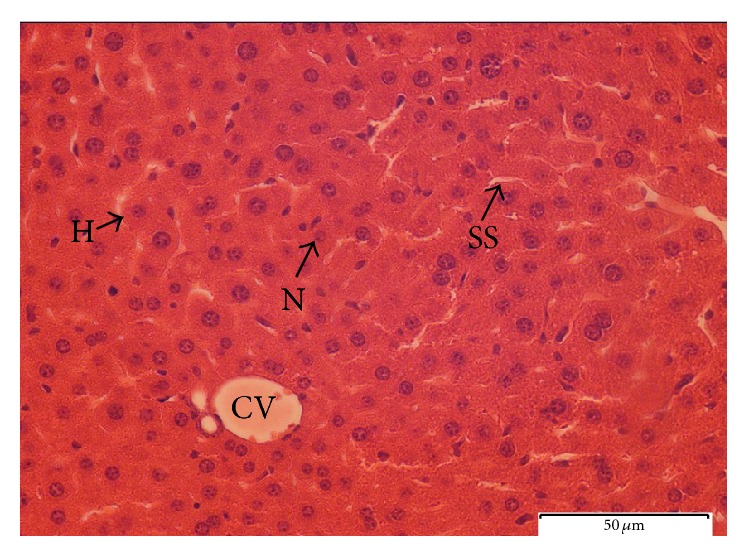
Light microphotograph of negative control liver. (H: hepatocytes; N: nucleus; SS: sinusoid; CV: central vein). Magnification: 40x.

**Figure 7:**
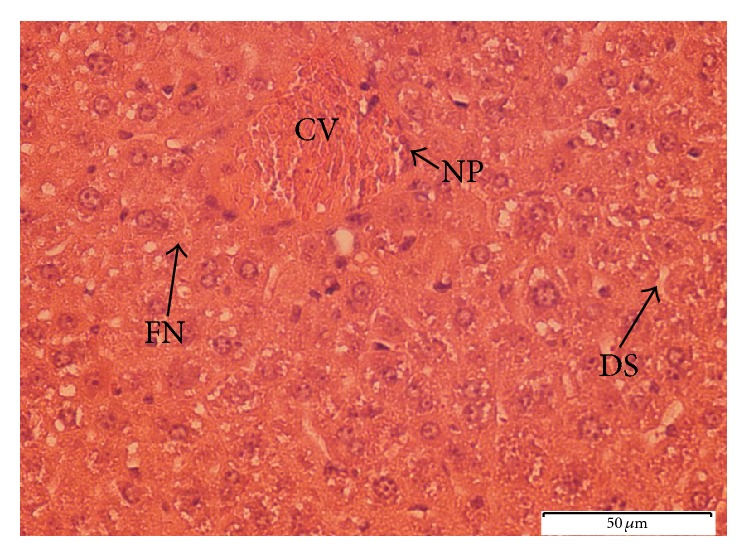
Light microphotograph of paracetamol-induced liver. (NP: neutrophil; DS: dilated sinusoid; FN: focal necrosis; CV: central vein). Magnification: 40x.

To further explore the biochemical analysis findings, histopathological observation was conducted on liver tissue. Liver sections taken from paracetamol-induced mice (Figure 6) had severe necrosis, vacuolar degeneration, loss of cellular boundaries, and obstruction of sinusoids, and hepatocytes were disrupted and showed hypertrophy compared to the healthy negative control group. The accumulation of neutrophils was also seen in the central vein. The neutrophils act as an indicator of the occurrence of cell damage as they are absent in normal healthy cells (Figure 7). Histopathological analysis showed that silymarin as well as the seed extract at a dose of 500 mg/kg b.w. significantly improved the degree of hepatocytes degeneration and necrosis in paracetamol-induced mice.

The hepatocyte nucleases are at a recovery stage and there are very minimal numbers of neutrophils surrounding the central vein.

**Figure 8:**
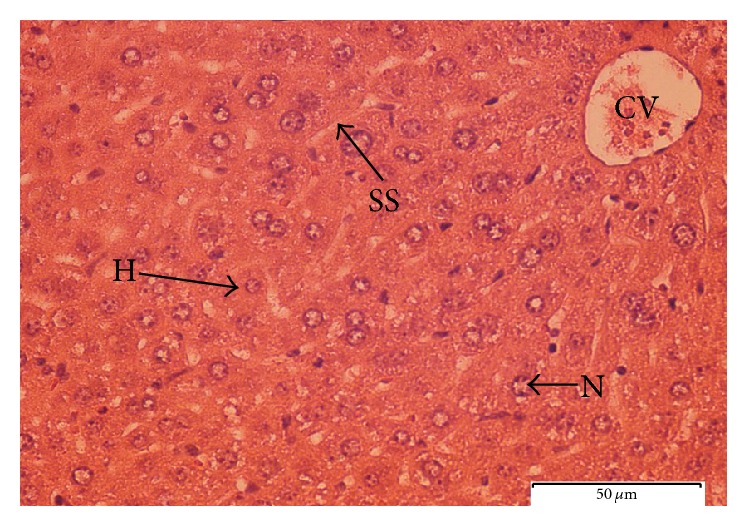
Light microphotograph of liver cells of mice treated with* C. surattensis *(250 mg/kg) (H: hepatocytes; N: nucleus; SS: sinusoid; CV: central vein). Magnification: 40x.

**Figure 9:**
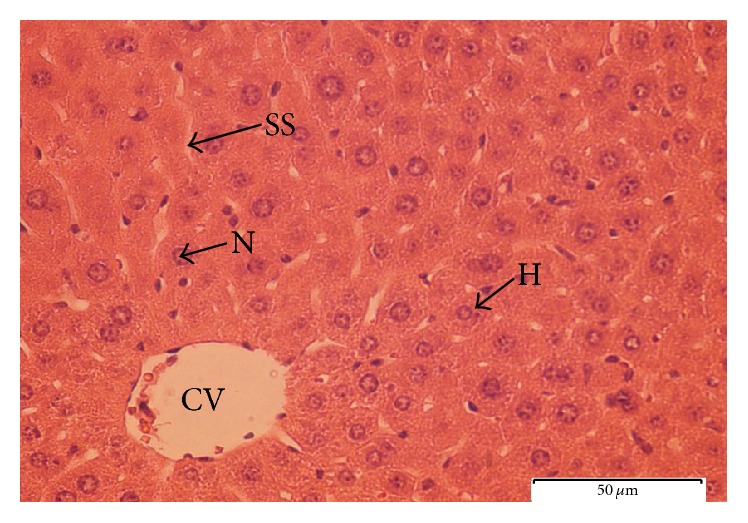
Light microphotograph of liver cells of mice treated with* C. surattensis *(500 mg/kg) (H: hepatocytes; N: nucleus; SS: sinusoid; CV: central vein). Magnification: 40x.

**Figure 10:**
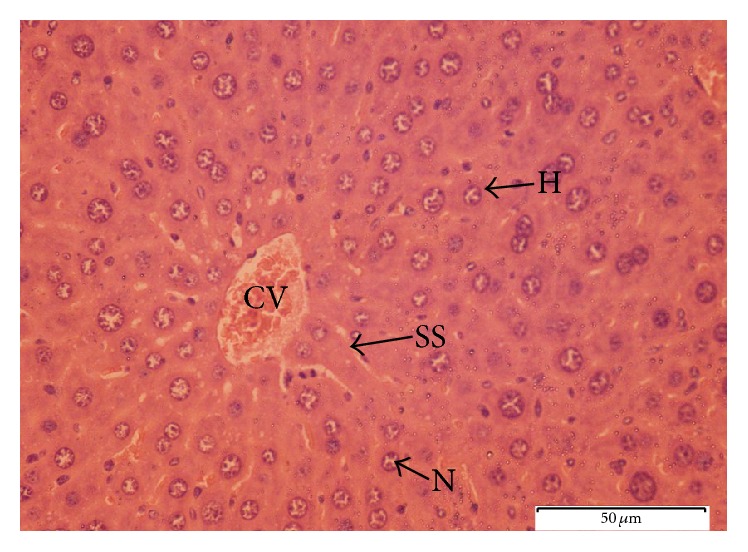
Light microphotograph of liver cells of mice treated with silymarin (H: hepatocytes; N: nucleus; SS: sinusoid; CV: central vein). Magnification: 40x.

The pretreated group with seed extract at a dose of 500 mg/kg b.w. (Figure 9) and silymarin at a dose of 200 mg/kg b.w. (Figure 10) was very close to the negative control group which showed intact liver cells. Histopathological liver sections of control group showed normal cellular structure and clear central vein, and hepatic cells were distinct and separated by sinusoidal spaces. The seed extract at a dose of 250 mg/kg b.w. did not show significant improvement over the effect of paracetamol on the liver (Figure 8). Though the extent of hepatocytes degeneration was a little lower than in the paracetamol-induced group, liver architecture (necrosis) was slightly improved.

## 4. Discussion

### 4.1. Total Phenolic Content

This study showed that* C. surattensis *seed extract contained favourable amount of phenolic compounds. A few studies have reported that* C. surattensis *plant parts possess a significant total phenolic content. For example, Sangetha et al. [24] reported that phenolic compounds were found in flower, stem, leaves, and pod of* C. surattensis* extracts and among the plant parts, only the flower, stem, and leaves of* C. surattensis* extracts showed a significant high content of total phenolics. Chew et al. [41] have reported that the flowers of* C. surattensis* extract contain total phenolic content of 3330 ± 309 mg GAE/100 g.

The result of the present study clearly indicated that the methanol extract of* C. surattensis* seed exhibited the presence of phenolic compounds and there was a strong positive correlation with the DPPH radical scavenging activity (*r* = 0.968) and NO radical scavenging activity (*r* = 0.979). The same relationship was also observed between phenolics and antioxidant activity in Sea Buckthorn extracts [42]. Kaneria et al. [43] reported that there are high correlation between phenolic contents and antioxidant activities of some medicinal plant extracts such as* Azadirachta indica, Hemidesmus indicus, Manilkara zapota, Psoralea corylifolia, Rubia cordifolia, *and* Tinospora cordifolia*. These show that the presence of a significant amount of total phenolic content in the seed extract might effectively inhibit radicals and contribute directly to the effective antioxidant activities. The phenolic compound in plant donates their hydrogen atoms from their hydroxyl groups to radicals and form a stable phenoxyl radical and this reaction contributes to their antioxidant activity [44].

### 4.2. Radical Scavenging Activity

#### 4.2.1. 2,2-Diphenyl-1-picrylhydrazyl (DPPH) Radical Scavenging Assay

The DPPH radical is regularly used as a substrate to evaluate the antioxidant activity of medicinal plant; it is a stable free radical that can receive an electron to become a stable molecule. The* C. surattensis* seed extract with higher concentration showed higher bleaching ability of the DPPH^•^ solution with greater hydrogen atom donating activity and higher antioxidant activity. The antioxidant activity of the extract might be attributed to the hydrogen donating ability of the phenolic hydroxyl groups [45, 46]. The DPPH scavenging activities of* C. surattensis* seed extracted with acetone have been reported in previous study by Deepak et al. [27]. According to the study, acetone extract showed maximum antioxidant activity, 76.11% than other extracts (methanol, chloroform) in comparison to standard drug, ascorbic acid.

#### 4.2.2. Nitric Oxide (NO) Radical Scavenging Assay

Nitric oxide (NO) is a key chemical facilitator involved in the regulation of various physiological processes. Higher concentration of NO is related with numerous diseases. Oxygen molecules react with the excess amount of NO to generate nitrite and peroxynitrite anions, which eventually act as free radicals [47]. The results of this study indicate that all the concentrations of* C. surattensis *seed extract and ascorbic acid tested have noticeable inhibition effect on nitric oxide radicals. The seed extract exhibited antioxidant activity through competing with oxygen to scavenge for the nitrite radical that was generated from sodium nitroprusside (SNP) at physiological pH in an aqueous environment. The* C. surattensis *seed extract acts as a potent antioxidant and donate protons to the nitrite radical. This decreased the absorbance and increased the percentage of inhibition. The decrease in absorbance and increase in percentage of inhibition were used to measure the extent of nitrite radical scavenging and antioxidant activity [48]. The NO scavenging activity of phenolic compounds is known [49–51], and this suggests that these compounds in the seed extract might contribute to the NO scavenging activity observed in this study.

#### 4.2.3. Reducing Activity

Reducing power assay is commonly used to assess the capability of a natural antioxidant to donate an electron [52]. This finding of this study affirms the reducing power of* C. surattensis *seed extract. It was hypothesized that the reducing power of* C. surattensis* seed extract might be due to their electron-donating ability and higher amount of reductants contains. Electrons or hydrogen atoms might react with free radicals to stabilize or transform the free radicals into more stable and nonreactive products subsequently blocking the free radical chain reactions [53, 54]. Therefore,* C. surattensis *seed extract could act as electron donors to free radicals and then dismiss the free radical chain reactions.

### 4.3. In Vivo Antihepatotoxic Activities

#### 4.3.1. Determination of Body Weight and Relative Liver Weight

The body weight of the mice in this study was not affected by the treatment of paracetamol, silymarin, and* C. surattensis* seed extract. This finding was parallel with the findings by Saad et al. [55] on the failure of thioacetamide to cause changes in body weight of rats in acute liver injury study. The increase in the liver weight and relative liver weight of paracetamol-induced liver hepatotoxicity group compared to negative control, silymarin and* C. surattensis* seed extract (doses of 250 mg/kg b.w. and 500 mg/kg b.w.), pretreated groups might be associated with the blocking of hepatic triglyceride secretion into plasma or might be due to the very large increase in liver hemoglobin [56]. On the other hand, the pretreatment with silymarin and seed extract prevented the increase in liver weight and relative liver weight when compared to paracetamol-induced group, which proved the significant hepatoprotective effect.

#### 4.3.2. Biochemical Analysis

Paracetamol induced mice liver damage in this study because of the formation of toxic metabolite, NAPQI, which is initially detoxified by conjugation with glutathione to form mercapturic acid [57]. The overdose of paracetamol used in this study caused the glutathione content of hepatocytes exhausted and the hepatocytes become vulnerable to the noxious effects of NAPQI resulting in liver cell necrosis or liver failure that eventually increased the ALT and AST levels [58, 59] which may explain the findings of this study. It was concluded that a single dose of paracetamol had caused damage to the parenchymal cell in the liver that leads to the leakage of the aminotransferases (ALT and AST) into the blood serum, resulting in increased of its concentrations. The aminotransferases that are released into the blood circulation when there is hepatic necrosis making the enzymes measurable in serum [60–62].

However, pretreatment of high dose of* C. surattensis *seed extract at 500 mg/kg b.w. and silymarin at 200 mg/kg b.w. exhibited an ability to counteract the toxic effect of paracetamol by decreasing the level of these enzymes. The pretreatment of* C. surattensis *seed extract at a dose of 250 mg/kg b.w. caused a slight decrease in the elevated serum liver enzymes. The results of this study supported the generally accepted view that serum levels of aminotransferases return to normal levels with the healing of hepatic parenchyma and regeneration of hepatocytes [63, 64]. The decrease in AST and ALT levels by the seed extract was also an indication of stabilization of plasma membrane as well as repair of hepatic tissue damage caused by paracetamol.

The pretreatment with the 250 mg/kg b.w. dose of seed extract slightly reduced the elevated serum liver enzyme levels but not as effective as the treatment with 500 mg/kg b.w. dose of the seed extract. This proved that* C. surattensis* seed extract at higher dose, 500 mg/kg b.w., showed a better hepatoprotective effect compared to the dose at 250 mg/kg b.w. This might be because the amount of antioxidant polyphenol compound(s) in the extract which render the free radical scavenging activity present at higher dose in 500 mg/kg b.w. compared with 250 mg/kg b.w. dose of* C. surattensis* seed extract. Interestingly, the in vitro antioxidant activity study showed that the* C. surattensis *seed extract exhibited a concentration dependent activity which supports this explanation.

This study also showed that in mice group with paracetamol-induced liver hepatotoxicity, the ALT and AST levels were elevated more compared to the lesser increase in the ALP level. This finding indicated that paracetamol caused cell necrosis at a single dose of 1 g/kg b.w. as cell necrosis involves the initial increase of ALT and AST and modest increase in ALP. The ratio of ALT/AST : ALP plays an important role in the determination of the types of liver damage by hepatotoxins. The ratio is greater than or equal to five for cell necrosis injury compared to if the ratio is less than or equal to two for cholestasis injury.

#### 4.3.3. The Antioxidant Status in the Liver Tissues

The ROS induced oxidative stress inside the cells triggered damage to hepatic parenchymal cells leading to hepatic injury [65]. The raise of ROS levels related with hepatic injury is due to high generation of ROS and reduced scavenging potential of the cells intrinsic antioxidants such as GSH and SOD [65]. Moreover, ROS may cause membrane damage by lipid peroxidation, and MDA is one of the final products of polyunsaturated fatty acids peroxidation in the cells [66]. In this study, paracetamol-induced liver hepatotoxicity significantly reduced the levels of SOD and GSH and elevated the level of MDA, which is a marker of lipid peroxidation, compared to negative control. The overproduction of NAPQI by overdose of paracetamol caused the glutathione content of hepatocytes exhausted and resulting in the reduction of GSH levels in liver tissue [58, 59]. The* C. surattensis* seed extract pretreatment was found increased in the level of intrinsic antioxidants such as GSH and SOD, with reduction in the elevated level of MDA, caused by the paracetamol-induced liver hepatotoxicity. Silymarin also caused significant favourable effects on the levels of GSH, SOD, and MDA relative to the paracetamol-induced group. These results propose that* C. surattensis* seed extract caused hepatoprotective activity linked with the improvement of in vivo antioxidant activity.

#### 4.3.4. Histopathological Observation

The methanolic extract of* C. surattensis* seeds showed significant hepatoprotective activity on paracetamol-induced liver toxicity when administered at a high dose of 500 mg/kg orally. The histopathological liver sections of* C. surattensis* seed extract (dose 500 mg/kg b.w.) pretreated group showed a comparable results to the silymarin pretreated group (dose 200 mg/kg b.w.) in recovering the paracetamol-induced histopathological lesions. These findings suggest the protection ability of 500 mg/kg b.w. dose of* C. surattensis* seed extract on membrane fragility and thus decreased the leakage of the serum liver enzymes into the blood circulation as observed in this study.

The seed extract which exhibited a concentration dependent activity was not effective in recovering histopathological lesions at a dose of 250 mg/kg b.w. as* C. surattensis* seed extract is more effective at higher concentration. The liver histopathological analysis was positively concomitant with the biochemical analysis of this study. The macroscopic appearance of the paracetamol-induced liver showed broad areas of necrosis that helps the leakage of the serum liver enzymes into the blood stream, and this explains the rise in AST, ALT, and ALP in the blood. The hepatotoxic effect of paracetamol is mainly due to generation of free radicals following the depletion of endogenous antioxidants such as glutathione [67]. Therefore, the herbal green* C. surattensis* seed antioxidant activity is effective in treating paracetamol-induced liver hepatotoxicity by scavenging the free radicals generated by the reaction of paracetamol.

In the present study, the* C. surattensis* seed extract was administrated at 250 mg/kg b.w. (low dose) and 500 mg/kg b.w. (high dose) based on the finding on acute oral toxicity of* C. surattensis* flower methanolic extract on Swiss albino mice. No sign of toxicity in mice was reported in the study conducted by Sumathy et al. [68]. According to the OECD guidelines for testing of chemicals 420 for testing of chemicals [69], animals should only be administrated a single dose of 5000 mg/kg. Hence in this study, the high dose of 500 mg/kg bw was selected as 1/10 of the dose that was used in the acute oral toxicity study (5000 mg/kg) and the low dose of 250 mg/kg b.w. was 2 times reduction of the high dose. Moreover, there were several studies that used doses of 250 mg/kg b.w. and 500 mg/kg b.w. to evaluate the hepatoprotective activity of plants [34, 70].

The methanolic extract of* C. surattensis* seeds in this study showed a good scavenging activity of DPPH and nitric oxide radicals. Hence, the in vitro findings further explained the potential of* C. surattensis* seed hepatoprotective activity in paracetamol-induced liver toxicity in mice and proved that the hepatoprotective activity may be due to the observed redox control of antioxidant activity of* C. surattensis* seeds. Similar hepatoprotective activity of* C. surattensis* leaf extract was also determined in the previous study designed to investigate the effect of ethanolic extract of* C. surattensis* leaf on CCl_4_-induced liver toxicity in rat. The results of the previous study suggested that* C. surattensis* leaf extract significantly reduced the elevated levels of AST, ALT, and ALP in serum which indicates the efficacy of the leaf extract as a redox control hepatoprotective agent [28]. These current histopathological findings further verified the redox control antihepatotoxic activities of* C. surattensis* seed extract in paracetamol-induced liver toxicity in mice model.

## 5. Conclusions

In conclusion, the results of the present study strongly suggested that* C. surattensis *seed has redox control of antioxidant and antihepatotoxic activities at lower dose by enhancing antioxidant activity and protecting the liver from the toxic effect of the hepatotoxic agents.

## Figures and Tables

**Table 1:** Mice groupings and administrated treatments.

Groups	Treatment
Negative control	10% DMSO
Induced	1.0 g/kg paracetamol per body weight
Treatment Group I	250 mg/kg seed extract per body weight + paracetamol
Treatment Group II	500 mg/kg seed extract per body weigh + paracetamol
Positive control	200 mg/kg silymarin per body weight + paracetamol

15 adult mice were divided into 5 groups (*n* = 3).

**Table 2:** Effect of *C. surattensis *seed extract on the body and liver weight of mice in paracetamol induced hepatotoxicity.

Groups	Dose (mg/kg)	Body weight, BW (g)	Liver weight, LW (g)	Relative liver weight (%) (LW/BW)
10% DMSO pretreated negative control	—	39.88 ± 3.15	2.32 ± 3.01	5.82 ± 0.31
Paracetamol-induced	—	38.99 ± 5.12^*∗∗*^	3.18 ± 5.01^*∗∗*^	8.15 ± 0.35^*∗∗*^
Silymarin + paracetamol	200.00	41.51 ± 3.12^*∗*^	2.54 ± 2.82^*∗*^	6.12 ± 0.41^*∗*^
Seed extract + paracetamol	250.00	39.85 ± 3.12^*∗*^	2.39 ± 3.08^*∗*^	5.99 ± 0.18^*∗*^
Seed extract + paracetamol	500.00	40.08 ± 3.48^*∗*^	2.42 ± 3.40^*∗*^	6.03 ± 0.47^*∗*^

Results are expressed in means ± SD (*n* = 3).

^*∗*^ *p* < 0.05 compared with paracetamol-induced group.

^*∗∗*^ *p* < 0.05 compared with 10% DMSO pretreated negative control group.

**Table 3:** Effect of *C. surattensis *seed extract on ALT, AST, and ALP (U/L) levels of mice in paracetamol-induced hepatotoxicity.

Groups	Dose (mg/kg)	ALT (U/L)	AST (U/L)	ALP (U/L)
10% DMSO pretreated negative control	—	16.72 ± 2.31	89.89 ± 4.12	114.3 ± 4.22
Paracetamol-induced group	—	1689 ± 102.14^a^	2998 ± 189.22^a^	341.51 ± 38.11^a^
Silymarin + paracetamol	200.00	601.1 ± 184.47^^ab^^	921.3 ± 298.21^ab^	185.47 ± 12.21^ab^
Seed extract + paracetamol	250.00	1105 ± 204.28^ab^	2031 ± 388.12^ab^	271.55 ± 27.32^ab^
Seed extract + paracetamol	500.00	801.43 ± 99.21^ab^	1389 ± 212.33^ab^	231.12 ± 11.17^ab^

Results are expressed in means ± SD (*n* = 3).

^a^ *p* < 0.05 compared with 10% DMSO pretreated negative control group.

^ab^ *p* < 0.05 compared with paracetamol-induced group.

**Table 4:** Effect of *C. surattensis *seed extract on oxidative status in paracetamol-induced hepatotoxicity mice liver tissue.

Groups	Dose (mg/kg)	GSH (mg/mg protein)	SOD (U/mg protein)	MDA (nmol/mg protein)
10% DMSO pretreated negative control	—	4.13 ± 0.12	120.82 ± 1.53	1.45 ± 0.11
Paracetamol-induced	—	2.35 ± 0.05	50.07 ± 1.22^*∗∗*^	5.04 ± 0.08^*∗∗*^
Silymarin + paracetamol	200.00	4.40 ± 0.04	136.69 ± 1.31^*∗*^	1.64 ± 0.01^*∗*^
Seed extract + paracetamol	250.00	3.80 ± 0.01	108.56 ± 3.51^*∗*^	1.87 ± 0.08^*∗*^
Seed extract + paracetamol	500.00	4.21 ± 0.09	130.34 ± 1.00^*∗*^	1.68 ± 0.05^*∗*^

Results are expressed in means ± SD (*n* = 3).

^*∗*^ *p* < 0.05 compared with paracetamol-induced group.

^*∗∗*^ *p* < 0.05 compared with 10% DMSO pretreated negative control group.
